# Mind(the)Plant: An expandable multimodal facility for the integrated characterization of plant behaviour

**DOI:** 10.1111/2041-210x.70411

**Published:** 2026-09-03

**Authors:** Valentina Simonetti, Bianca Bonato, Silvia Guerra, Sara Avesani, Luca Semenzato, Maria Bulgheroni, Gabriela Gjinaj, Laura Ravazzolo, Marco Dadda, Umberto Castiello

**Affiliations:** 1 Department of General Psychology, University of Padova, Padova, Italy; 2 Ab.Acus Srl, Milan, Italy; 3 Department of Agronomy, Food, Natural Resources, Animals and Environment (DAFNAE), University of Padova, Legnaro, Italy

**Keywords:** kinematics, multimodal investigation, phytotron, plant behaviour, rhizocameras, vocs

## Abstract

1. Understanding plant behaviour requires the integration of multiple phenotypic and physiological signals measured over time under controlled conditions. However, different plant signals are typically studied using separate experimental setups, limiting temporal alignment and integrative analyses.
2. We present Mind(the)Plant, a modular experimental facility designed for the synchronized, long-term acquisition of multimodal plant data, including three-dimensional shoot kinematics, above- and below-ground volatile organic compounds (VOCs) and root imaging. Its modular architecture is designed to accommodate additional acquisition modules, such as electrophysiological signalling, as future extensions. The platform integrates a controlled growth environment with stereovision imaging, high-resolution time-of-flight mass spectrometry and custom rhizocameras. These components are connected through a unified network infrastructure that ensures synchronized acquisition and centralized data handling.
3. We validate the performance of each acquisition module through multi-week recordings, demonstrating high-temporal stability, reliable stereovision synchronization, effective isolation of VOCs signals and robust operation of below-ground imaging. We further illustrate the analytical potential of the platform using a one-day continuous multimodal acquisition combining shoot kinematics, above-ground VOC emissions, rhizocameras observations and environmental data.
4. Mind(the)Plant provides a novel methodological framework for studying plant behaviour, signalling and phenotypic plasticity in ecological and evolutionary research. By enabling coordinated measurements of multiple plant response modalities, the platform supports investigations of dynamic plant-environment and plant–plant interactions from a behavioural perspective.

## 1 Introduction

In recent years, interest in plant behaviour has grown substantially, supported by theoretical frameworks that conceptualize plants as organisms capable of deploying flexible strategies to cope with environmental challenges (Baluška & Levin, 2016; Bianchi et al., 2025; Trewavas, 2009). In this perspective, behaviour can be broadly defined as ‘all the actions directed by organisms toward the outside world in order to change conditions therein or to change their own situation in relation to these surroundings’ (Piaget, 2013). In plants, such behaviour emerges from coordinated physiological, biochemical and morphological processes that enable adaptation to heterogeneous and dynamically changing conditions (Karban, 2008; Novoplansky, 2009; Silvertown & Gordon, 1989).

In this context, major areas of investigation for the study of plant behaviour are the characterization of goal-directed and social behaviour through motion analysis of shoots and roots (Bonato et al., 2023, 2024, 2025; Ceccarini et al., 2020; Ciszak et al., 2012; Guerra et al., 2019, 2024; Migliaccio et al., 2013; Simonetti et al., 2025; Wang et al., 2023), plant signalling and communication through volatile organic compounds (VOCs) (Bergman et al., 2025; Dudareva et al., 2006; Karban et al., 2014; Kessler et al., 2023), and mechanisms of information processing and systemic signalling inferred from plant electrophysiological responses (de Toledo et al., 2019; Debono & Souza, 2019; Felle & Zimmermann, 2007; Parise et al., 2021, 2023; Vodeneev et al., 2016). These channels, however, represent only part of a much broader signalling repertoire. Plants also exchange information through additional chemical and physical cues including non-volatile root exudates, small RNAs, hydraulic and mechanical signals, and plant-emitted ultrasound (Dobránszki et al., 2025; Wang et al., 2021) that operate across different spatial and temporal scales. A complete characterization of plant behaviour would ultimately require integrating this full diversity of signals; the present work focuses on shoot and root motion together with VOCs emissions as a first, extensible step in this direction.

Different modalities are typically investigated in isolation using independent experimental setups. This fragmentation limits the ability to capture how different signalling channels interact across spatial and temporal scales, constraining the study of plant behaviour as an integrated process. Because plant responses emerge from the coordination of multiple processes operating simultaneously, understanding plant behaviour requires experimental setups capable of synchronizing and integrating heterogeneous data streams. The lack of such systems represents a major methodological limitation in current plant behavioural research (Bianchi et al., 2025).

Recent developments in computational phenomics and artificial intelligence, together with the increasing availability of computational infrastructures capable of handling big amounts of data, have highlighted the potential of multimodal data integration to improve the characterization and prediction of plant traits, enabling improved monitoring of plant health and agricultural productivity (Harfouche et al., 2023; Maimaitijiang et al., 2020; Streich et al., 2020; Tardieu et al., 2017). However, existing high-throughput phenotyping platforms have primarily focused on combining imaging modalities and environmental sensing for trait prediction (e.g. Li et al., 2021; Xu et al., 2026). The incorporation of VOCs monitoring into such frameworks is still very limited and technically challenging (Hall et al., 2022). These limitations are even more pronounced in studies specifically targeting plant behaviour, where truly multimodal and synchronized approaches are still rarely implemented (Bianchi et al., 2025).

To address this gap, we developed Mind(the)Plant, a new experimental facility designed for the synchronized, long-term acquisition of multimodal plant data, including three-dimensional (3D) shoot kinematics, VOCs emissions and root imaging. Its modular architecture is designed to accommodate additional acquisition modules, such as electrophysiological signalling of the aerial part of the plant, as future extensions. The facility integrates a controlled growth environment with a unified network infrastructure and centralized data architecture that allows the integration of complementary data streams through unified experimental pipelines.

In this manuscript, we describe the technical design of the Mind(the)Plant facility and validate the performance of its acquisition modules through targeted tests and multi-week recordings. We further present data from 1 day of simultaneous acquisition of all the modules and discuss how synchronized multimodal data acquisition enables integrative analyses of plant behaviour that are not achievable using isolated measurement systems. The incorporation of electrophysiological recordings is discussed as a planned extension of the facility.

## 2 Materials and Methods

### 2.1 Description of the facility

**Figure 1:**
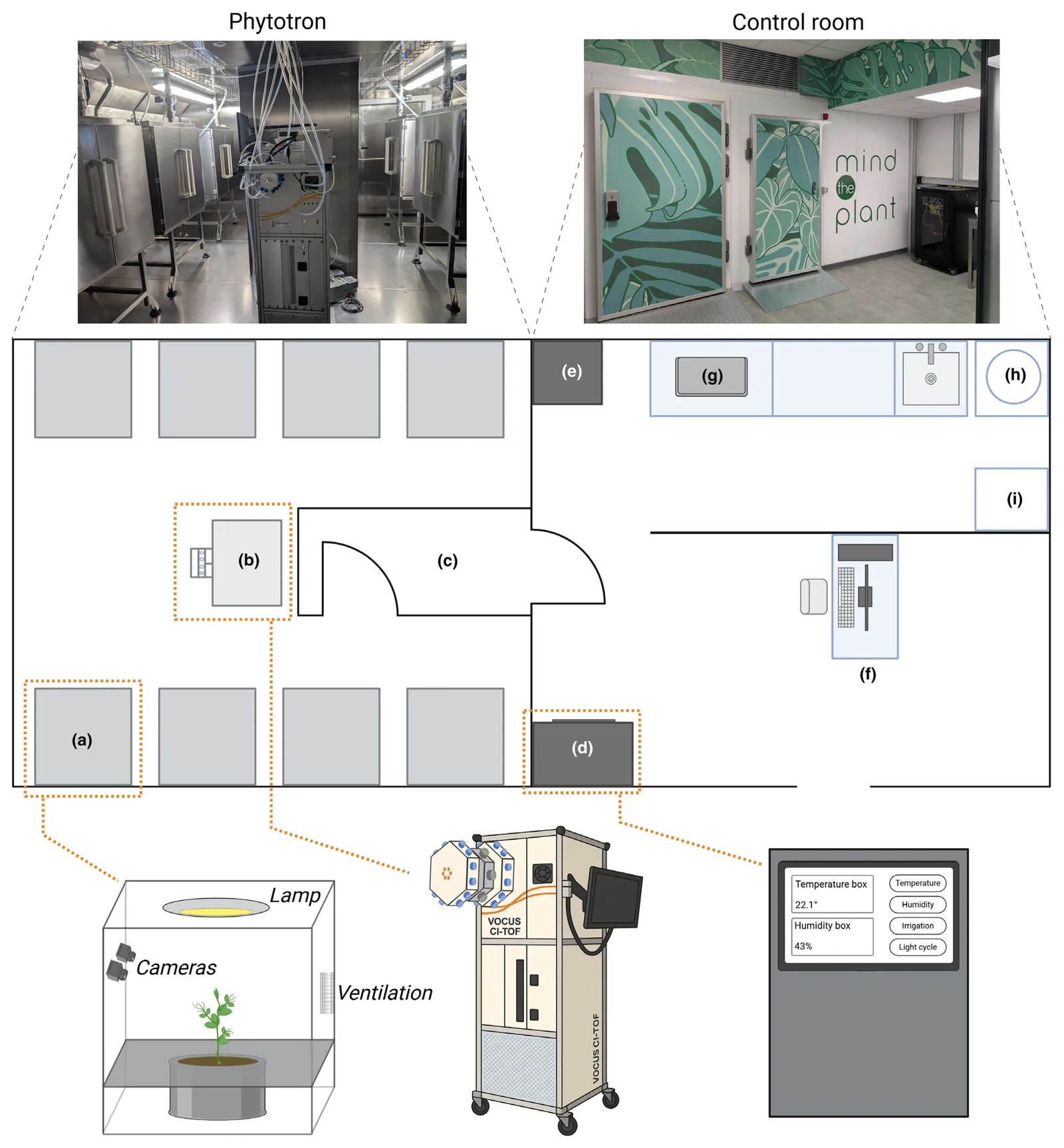
Mind(the)Plant laboratory equipment. (a) Growth box equipped with a pair of Power-over-Ethernet (PoE) 4K UHD RGB–infrared cameras working in stereovision, a cool white LED lamp and a ventilation system. (b) High-resolution Time-of-flight Mass Spectrometer (TOF-MS, Vocus 2R; Tofwerk AG, Thun, Switzerland) equipped with a multiport valve mounted at the inlet of the MS to manage parallel acquisition from multiple growth boxes. (c) Anteroom with lab coat hanger. (d) Operator panel connected to PLC logic for environmental settings. (e) Rack where all the Ethernet cables from the phytotron converge containing router switches and a managed USB hub. (f) User workstation. (g) LA2400 scanner (Epson Expression 13000XL, Seiko Epson Corporation, Suwa, Japan) for roots analysis (WinRHIZO, Regent Instruments, Québec, Canada). (h) Autoclave (STERISTEAM 2). (i) −86° ultra-low-temperature freezer (Smeg, Guastalla, Italy).

The Mind(the)Plant laboratory consists of two main areas: a phytotron and a control room (Figure 1).

**Figure 2:**
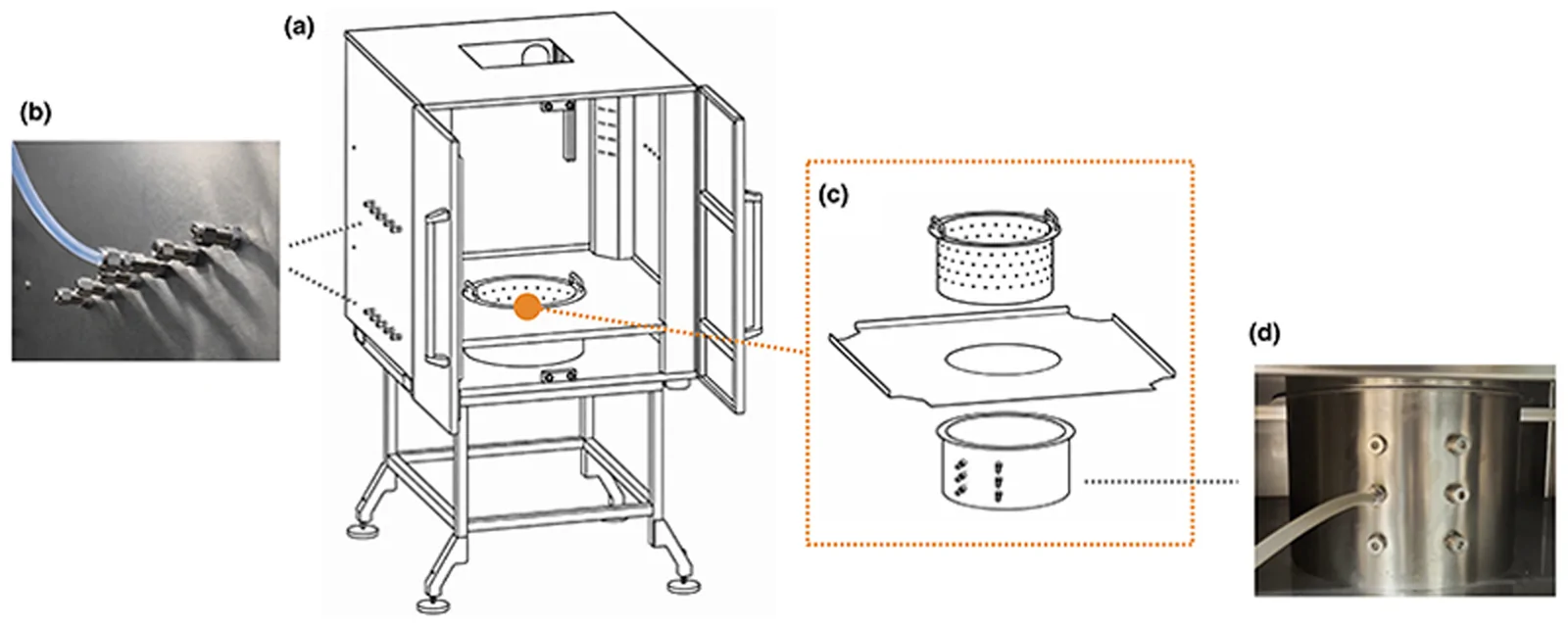
Growth box structure. (a) Schematic of the growth box showing the extractable pot positioned on the internal platform. (b) Photographic details of the Swagelok connectors integrated into the external walls for PFA tubing attachment. (c) Exploded view of the custom-made pot assembly, including the perforated upper container, interchangeable support plate and lower collection chamber. (d) Photograph of the pot with side connectors enabling below-ground VOCs collection.

The phytotron houses eight growth boxes and a high-resolution time-of-flight mass spectrometer (TOF-MS). Each box has a volume of 1 m^3^ (1 m × 1 m × 1 m) and is made of stainless steel 15/10. A horizontal partition separates the upper compartment in which the aerial part of the plants develops from a lower compartment housing the pots (Figures 1a and 2). The internal support plate on which the pot rests is interchangeable, allowing different pot configurations to be accommodated within the same growth box. In addition to the single-pot configuration shown in Figure 2, alternative plates can host a larger pot or two separate pots, enabling experiments on plant–plant interactions between individuals sharing the same controlled environment.

**Figure 3:**
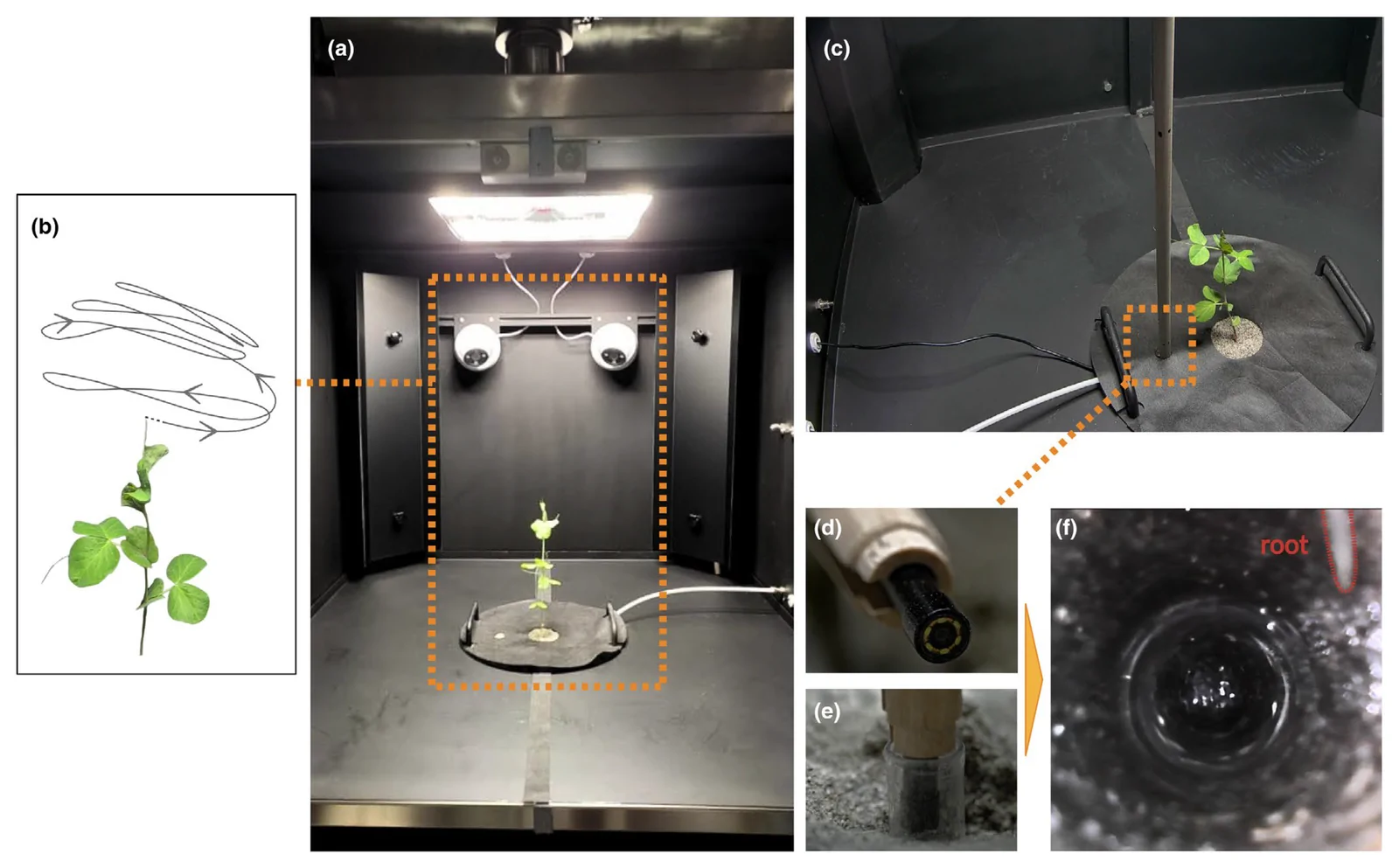
Growth box view with detail of custom cameras. (a) Front view of an open growth box showing the positioning of the pair of RGB–infrared cameras for motion reconstruction relative to a sample pea plant; a disc of non-woven permeable fabric is placed on the substrate surface to provide a uniform dark background for imaging and a reference for plant positioning. (b) Representation of the 3D trajectory of the plant tendril tip obtained. (c) Sample view from one of the two cameras showing another sample pea plant growing close to a wooden pole serving as potential climbing support housing the endoscope, used as custom rhizocamera. (d) Close-up of the endoscope. (e) Insertion of the endoscope into a transparent tube embedded in the substrate to work as a custom rhizocamera. (f) Representative root image captured by the custom rhizocamera where the root is highlighted in red.

The upper compartment of each box is equipped with a pair of Power-over-Ethernet (PoE) 4K UHD RGB–infrared cameras (RLC-833A, Reolink, Hong Kong, China; Figures 1a and 3a) mechanically mounted on a DIN rail that allows adjustable positioning. Each camera is powered and connected via Ethernet cable to a router switch (UniFi Pro 24-Port, Ubiquiti Networks, New York, NY, USA), which links them to the laboratory’s internal network. The pair of cameras works in stereovision to allow for three-dimensional (3D) plant motion reconstruction (Figure 3b).

Illumination is provided by a cool white LED lamp (Q150W v2.0, PURE LED, Spain) positioned at the top of each box (Figures 1a and 3a). Temperature, humidity, irrigation, lighting and air recirculation are automatically controlled within each box. Air quality and environmental stability are ensured by an air treatment unit comprising a prefilter, a filter, a fan and a steam humidifier (Esse Costruzioni S.r.l., Frosinone, Italy; Figure 1a). This system guarantees continuous recirculation of purified air and maintenance of the set environmental parameters. When closed, each box operates under slight positive pressure (Δ = 5 Pa, inside relative to outside), maintained by continuous airflow.

Each box is also equipped with cable glands on the side to accommodate USB industrial endoscopes (Depstech 86 T 100w, Shenzhen, China), which are used as custom rhizocameras. Each rhizocamera is connected to the Ethernet via a USB-to-Ethernet extender (EX-1441-2, Exsys Italia srl, Como). These rhizocameras are intended for experiments involving static subsurface elements that may be encountered by roots (Figure 3c–f).

The high-resolution TOF-MS (Vocus 2R; Tofwerk AG, Thun, Switzerland; Figure 1b) can support two interchangeable reactors: a Proton Trasfer Reaction (PTR) reactor and an Adduct Ionization Mechanism (AIM) ion-molecule reactor. The inlet to the reactor of the MS is connected to a multiport valve (Tofwerk AG, Thun, Switzerland; Figure 1b) that enables the parallel connection of multiple sampling lines. Each growth box is associated with a dedicated valve port of the multiport using perfluoroalkoxy (PFA) Teflon tubing (length: 6 m; outer diameter 3/8 in). The box is mechanically tied to the tubing through Swagelok connectors integrated into the box’s external walls (Figure 2b). The pots were custom-made to be extractable and to enable the monitoring of below-ground VOCs through openings incorporated into their structure (Figure 2c,d). Above- and below-ground VOCs signals are acquired independently through dedicated lines. The two compartments are structurally separated by the horizontal partition (Figure 2c). The below-ground line directly samples from the pot support. A limited air exchange between compartments through the substrate cannot be excluded.

Because the facility uses a single high-resolution TOF-MS served by a multiport valve, two operating modes are supported. In the multiport mode, the valve cycles through all boxes, providing sequential VOCs monitoring across all the boxes at a reduced per-box temporal resolution (each box is sampled once per cycle). In the dedicated mode, the TOF-MS samples a single box continuously, providing high-temporal resolution (up to 10 Hz). The multiport is automatically controlled by a cycling table that can be programmed by the user to manage the serial switches from one box to the other. In this way, the cycling table can be adapted to the experimental design. The cycling table is designed to continue the cycles of iterations indefinitely until the operator stops the acquisition.

The TOF-MS can cover days-to-weeks acquisitions, operating in a quasi-continuous regime: It requires brief, scheduled interruptions of a few minutes for periodic mass and sensitivity recalibration (performed weekly), which is the main practical constraint on strictly continuous operation. Other maintenance operations do not constrain acquisitions on this timescale: The reagent is replaced every 5–6 months under intensive use, and spare-parts replacement and periodic checks are performed on a yearly basis.

The control room is separated from the phytotron by an ante-room equipped with a lab coat hanger (Figure 1c). The main function of the control room is to manage samples and monitor ongoing experiments without accessing the phytotron and opening the boxes. A touchscreen panel connected to a programmable logic controller (PLC, Esse Costruzioni S.r.l., Frosinone, Italy; Figure 1d) allows the user to set and adjust the experimental growth parameters, including temperature, humidity, irrigation frequency, fertilizer proportion, total irrigation volume and light–dark cycle. The control room is also equipped with different instrumentation for sample preparation and measurement.

A dedicated equipment rack collects all Ethernet connections from the phytotron (Figure 1e). Some Ethernet cables provide power and data transfer for the PoE cameras inside the growth boxes. These cables connect to a UniFi Pro 24-Port router switch (Ubiquiti Networks, New York, NY, USA), which links to the laboratory’s internal network. Other Ethernet cables from the phytotron carry image data from custom rhizocameras and connect to an Ethernet-to-USB reverse adapter. This adapter is linked to a managed USB hub (EX-7570HMVS, Exsys Italia srl, Como), which connects to the workstation through a USB extender (EX-1405, Exsys Italia srl, Como).

The workstation (Figure 1f) enables real-time monitoring of the running experiments and manages the acquisition of images from both the cameras inside the growth boxes and the rhizocameras. Being connected to the same network, the workstation has continuous access to the camera view and settings. The rhizocameras are connected to the workstation via the USB extender from the USB hub. The workstation runs custom software for controlled image acquisition from the cameras and the custom rhizocameras at user-defined frame rates (ROOMors-Camrecorder, Ab.Acus s.r.l., Milan, Italy).

Temporal synchronization between data collected by the MS and the workstation is ensured by automatic network time synchronization: Both the MS computer and the workstation are connected to the internet and use the Network Time Protocol (NTP) for clock alignment.

**Figure 4:**
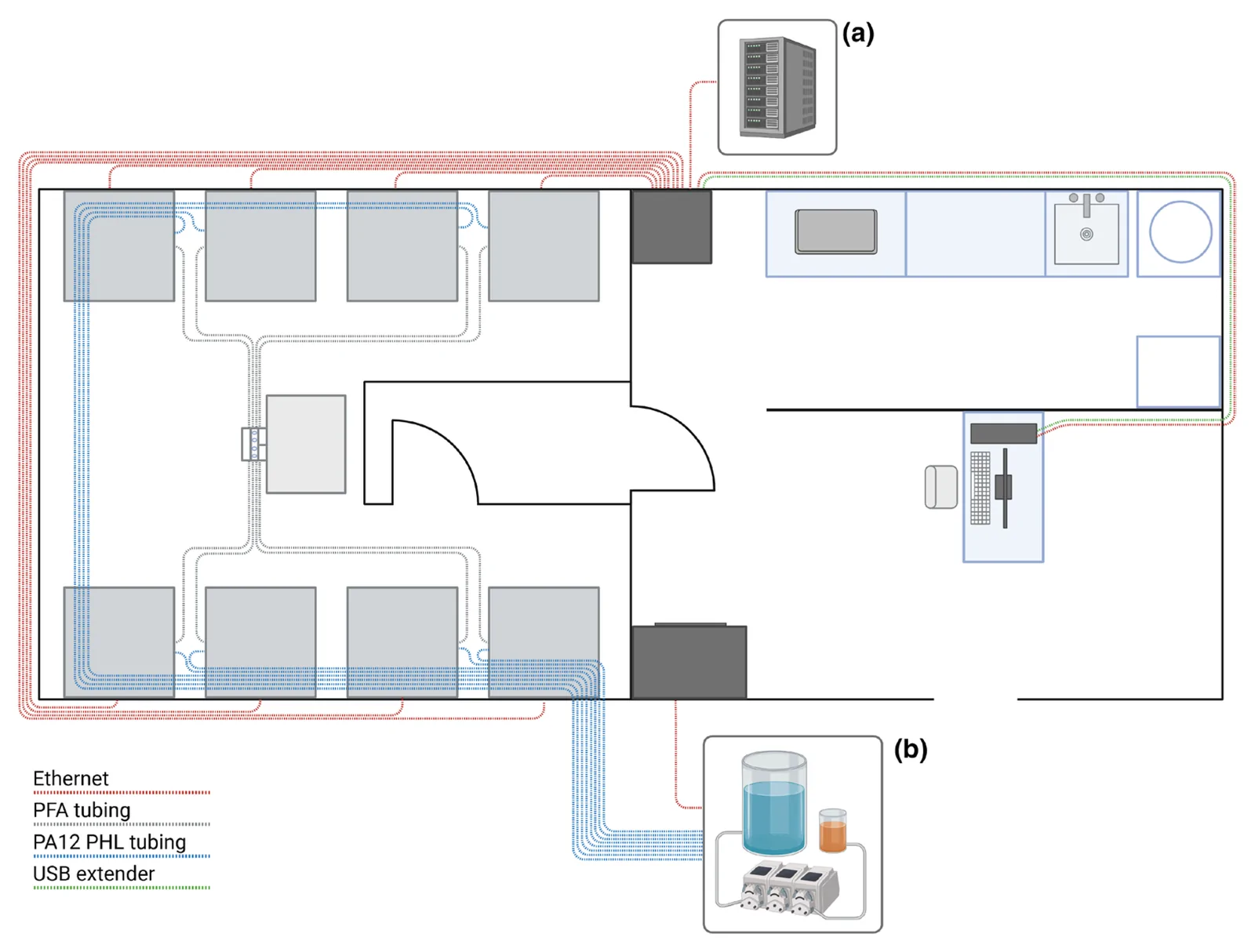
Mind(the)Plant laboratory connections. Connections between elements inside the Mind(the)Plant laboratory that guarantee the correct functioning of the laboratory. Each growth box has ethernet connections that are used to power and connect the cameras and the rhizocameras. All the ethernet cables go inside the rack in the control room where they are connected to the router switch (for cameras) and the managed USB hub (for rhizocameras). The workstation is connected via ethernet to the internal network and collects the USB cable from the USB hub used for the rhizocameras. Perfluoroalkoxy (PFA) tubing is used to connect each box with the inlet of the MS through the multiport. PA12 PHL tubing carries the water solution used for irrigation in each box. (a) Datacentre connected to the laboratory network and physically running in an adjacent room. (b) Irrigation solution preparation system, physically running in the floor above the laboratory.

Following best practices for the effective management of plant digital phenomics and computational phenomics (Harfouche et al., 2023), we designed and implemented a dedicated IT infrastructure, data architecture and set of tailored data processing pipelines. All the devices are connected through a network infrastructure that ensures high-throughput data transfer, communication among devices and controlled access through a firewall gateway. All the data streams converge on a dedicated virtualized datacentre infrastructure (Figure 4a). The system is based on enterprise-grade servers (2 blades cluster—Model: HP ProLiant DL380 Gen10 Plus—Processor Type: Intel(R) Xeon(R) Gold 5317 CPU @ 3.00GHz—Logical Processors: 48—Memory: 512 GB) running virtual machines that support data acquisition, storage and processing workflows.

**Figure 5:**
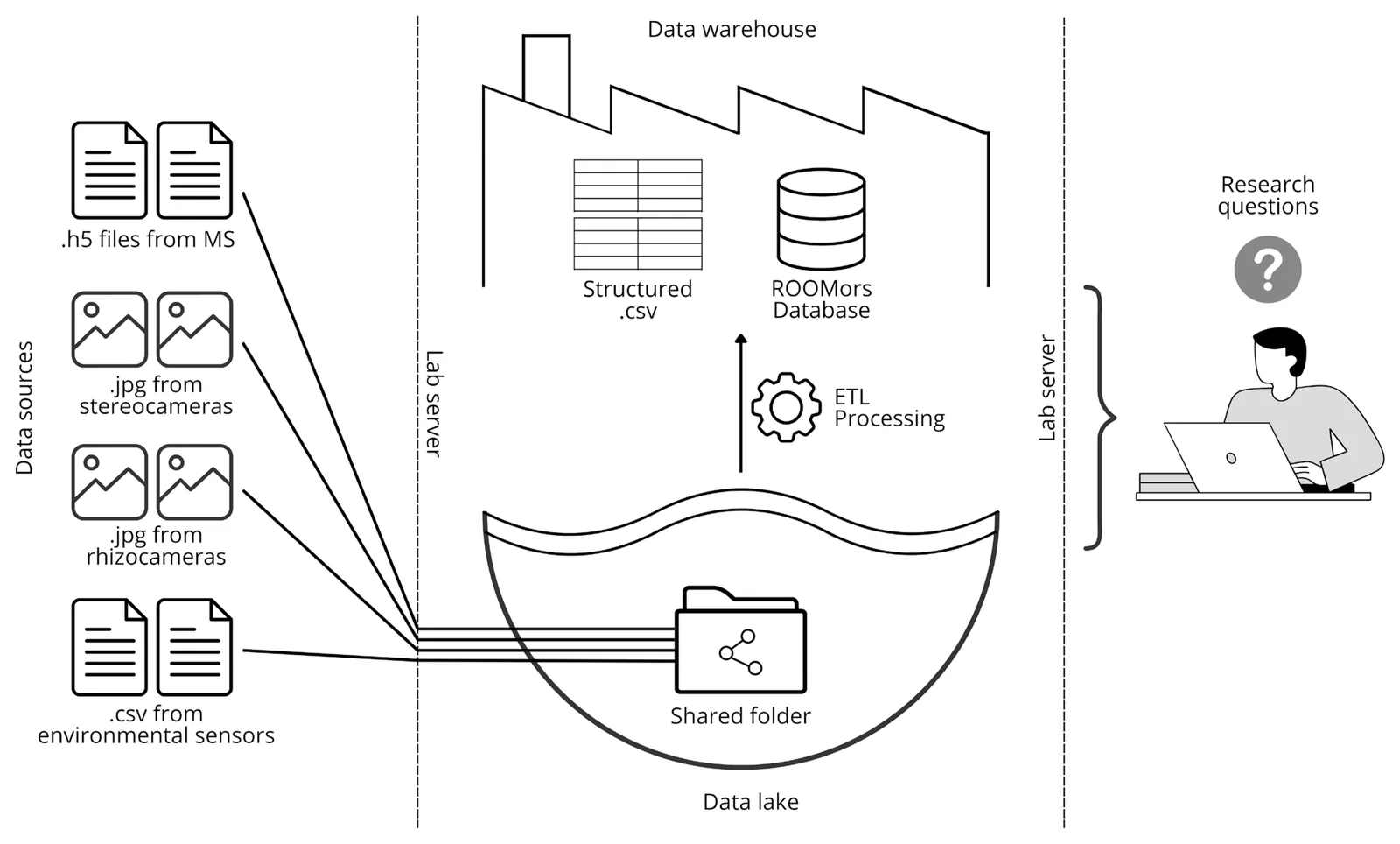
Mind(the)Plant data architecture. Unstructured data generated from the different data sources are saved in a shared data folder directly from the device running the acquisition process which is inside the same network of the laboratory server. In this way, raw data are directly saved in the data lake and are ready for pre-processing and ETL. Processed data generate structured .csv files and new entries in the ROOMors SQL relational database. In this way, generated data are available to support research investigation.

A terminal server farm provides centralized computational resources for data analysis that allows the users to perform intensive analyses relying on local workstation resources, ensuring scalability and reproducibility of data processing pipelines. The data architecture is reported in Figure 5. Unstructured and semi-structured data generated by the different data sources (VOCs measurements from the MS, shoot motion data from stereocameras, root imaging from rhizocameras and environmental sensor data) are directly stored in a shared folder exposed by a shared file server, which functions as a centralized data lake. Through Extract-Transform-Load (ETL) procedures and dedicated data processing pipelines, the unstructured data are converted into structured format, including .csv files and entries of the laboratory SQL database, which together constitute the logical data warehouse of the system. The ETL and processing pipelines rely on a combination of automatic and semi-automatic software tools and scripts including Tofware (Tofwerk AG, Thun, Switzerland), ROOMors-tracking (a graphical evolution of Sprouts software by Ab.Acus s.r.l., Milan, Italy (Simonetti et al., 2021)) and custom Python scripts for data parsing, processing and loading. This data architecture enables systematic and reproducible data exploration and supports the efficient integration and analysis of multiple high-dimensional data sources, thereby facilitating robust investigation of complex research questions in plant behaviour and phenomics.

**Figure 6:**
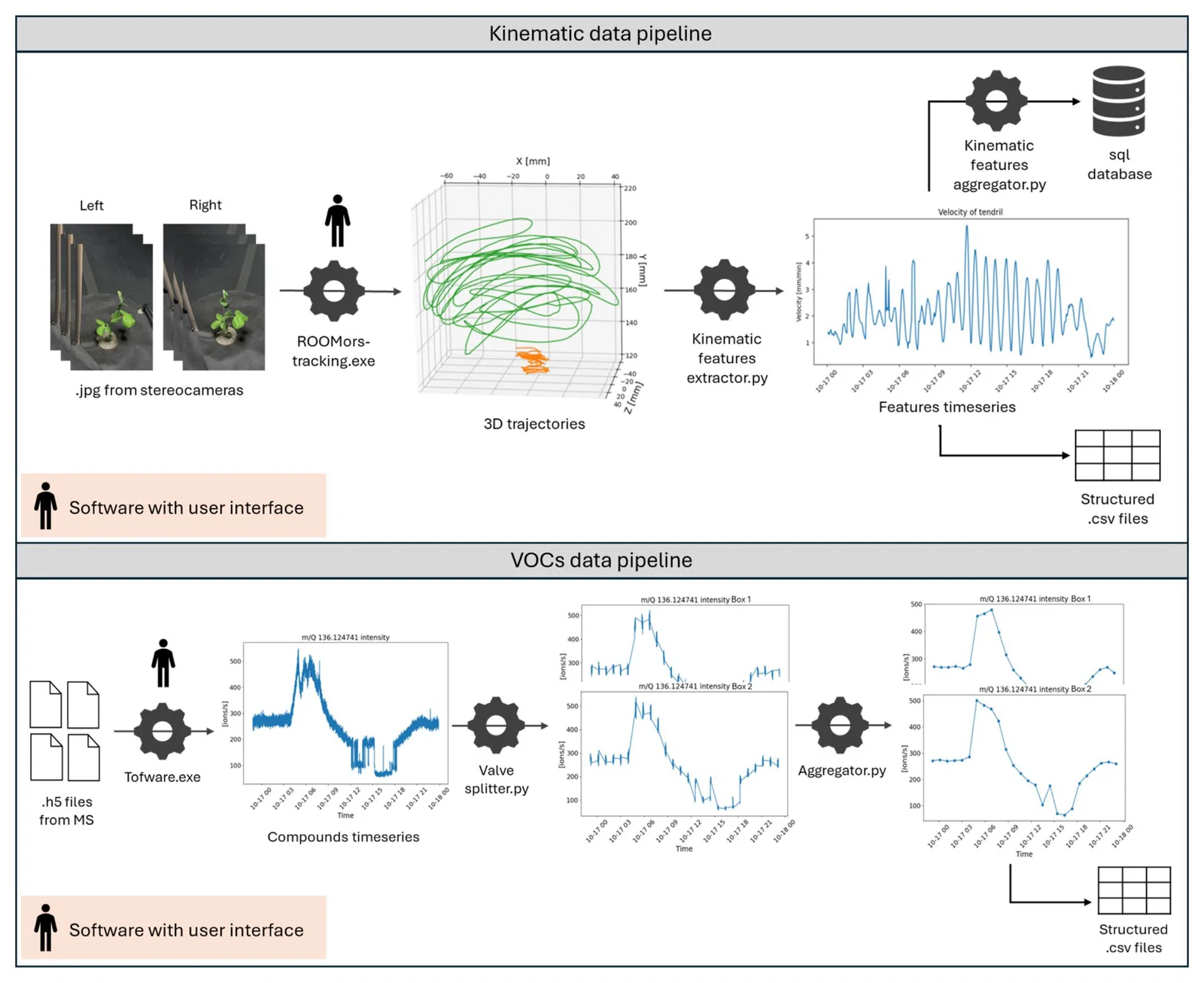
Overview of the kinematic and VOCs data processing pipelines. The upper panel illustrates the kinematic data pipeline: Raw stereo image sequences are processed through a proprietary tracking software (ROOMORS-tracking; Ab.Acus srl, Milan, Italy) to reconstruct 3D plant trajectories, which are then analysed to extract kinematic features (e.g. velocity, acceleration) both as time series and aggregated values. The lower panel shows the VOCs data pipeline: Raw mass spectrometry signals obtained with the Vocus 2R are processed using proprietary software (Tofware; Tofwerk AG, Thun, Switzerland) and applying an untargeted analysis to obtain time series of ion intensities, which are subsequently split according to valve switching and aggregated into summarized VOCs features. Both pipelines combine automated processing steps (gear icons) with user-interactive software components (human icon), ultimately producing structured datasets for downstream analysis and integration.

Figure 6 shows both the kinematic and VOCs processing pipelines from raw data to feature extraction. Images acquired with rhizocameras are visually inspected to identify the time when the root enters the endoscope’s field of view; this time is then manually recorded by the user. Because images are acquired at discrete intervals, the annotated time corresponds to the acquisition window between two consecutive frames in which root entry occurred. Environmental data are directly stored as structured .csv files.

For 1 week of full mode acquisition, the system collects around 30 GB of MS data and 25 GB of images from stereocameras and custom rhizocameras. A high-speed storage layer based on Solid-State Drive (SSD) and Non-Volatile Memory Express (NVMe) technology (≈10 TB) is dedicated to active experiments and real-time data processing. A second storage layer based on high-capacity rotational disks (≈50 TB) is used for long-term data preservation. This layer stores completed experiments and datasets that are not frequently accessed but must remain available for reproducibility and future analyses.

To ensure operational continuity during long-term experiments, the infrastructure is designed with multiple layers of logical redundancy. At the laboratory level, network connectivity is distributed across two independent switches, each hosting approximately half of the connected devices. This configuration ensures that, in the event of a switch failure, only a subset of devices becomes temporarily unavailable. Full system functionality can be restored by physically reconnecting affected devices to the remaining operational switch. Redundancy is further implemented at the uplink level between the rack and the datacentre. The laboratory network is connected through a logical trunk composed of dual physical links, each terminating on separate core switches within the datacentre. These core switches operate in a logically redundant configuration and are cross connected to the server infrastructure, ensuring continuous data flow even in the presence of a single link or switch failure. At the computing layer, redundancy is ensured through virtualization technologies. The infrastructure relies on a high-availability orchestration system (VMware), which automatically migrates virtual machines from a failing physical host to a healthy node in case of hardware failure. This mechanism guarantees service continuity for acquisition, storage and processing pipelines without manual intervention.

All virtual machines and storage volumes are protected through automated incremental backup procedures managed by a centralized backup system (Veeam), with replication to dedicated storage units. Backup policies are applied dynamically based on system tagging, ensuring consistent protection across all components of the infrastructure.

Electrical continuity of the whole system is ensured through a layered backup architecture. Servers, cameras and rhizocameras are protected by a dedicated single-phase (3.5 KW) uninterruptible power supply (UPS) providing instantaneous switchover with no interruption with estimated backup time of 4 h. The phytotron systems, including MS, ventilation, lighting and irrigation, are supported by a separate three-phase UPS (3.5 KW) able to sustain the controlled environment for at least 4 h. Both UPS units are connected downstream of a building-level diesel generator providing continuity for an unlimited duration. The three-phase UPS is dimensioned to cover the generator’s start-up latency, ensuring a seamless transition during a power outage.

### 2.2 Validation of VOCs data acquisition pipeline

To validate the VOCs data acquisition pipeline, we verified that each growth box, when closed and maintained under positive pressure by continuous airflow, was effectively isolated from external contamination. In addition, we assessed whether the multiport valve correctly and selectively sampled air from the different growth boxes. A commercial essential oil containing linalool (Lavande vraie, Aroma Zone, Cabrières-d’Avignon, France) was used as a known, controllable volatile signal. This commercial oil was used as a reproducible source of linalool rather than as a quantified standard. The validation therefore assessed signal detection, isolation from external contamination and multiport selectivity, not absolute VOC quantification. Validation was performed through these acquisition runs:

1. Baseline acquisition:
  Five complete acquisition runs (systematic sampling from box 1 to box 8 using the multiport valve with the cycling table) were performed in the absence of any volatile source to establish the baseline VOCs signal.
2. External-contamination control:
  Five complete acquisition runs were conducted with the essential oil source open inside the phytotron but outside the growth boxes. This test evaluated whether linalool (m/Q 154.134979) present in the external environment could be detected inside the boxes, thereby assessing isolation from external contamination. Baseline acquisitions and external-contamination controls were alternated.
3. Plateau reaching and decay test:
  One complete acquisition run was performed using this protocol for each growth box:
  - 30s with the box empty.
  - Essential oil source open inside the box until a plateau was reached.
  - Essential oil removed and box left empty until the linalool signal reached values comparable to the initial (mean + 3 SD of baseline).
  This acquisition was used to obtain reference values of intensity when the linalool source is placed inside the box and to assess the time of recovery for each box when the source is removed.
4. Selective sampling test:
  Two complete acquisition runs were performed using the following protocol for each growth box:
  - 20 s with the box empty.
  - 20 s with the essential oil source open inside the box.
  - 40 s with the box empty.
  This acquisition tested the ability of the multiport valve to selectively sample individual boxes and verified that the expected linalool signal was correctly detected by the MS. The essential oil was introduced by briefly opening the box and placing the source inside. To test that the multiport correctly and selectively samples each box, we analysed the time series collected. To validate the system, we expected the measured signal corresponding to the linalool channel to start increasing 20 s after the valve switched to the box—the moment when the essential oil source is opened and linalool is released inside the box—followed by a negative step at 80 s, when the multiport switched to the next box. Between 40 and 80 s, we expected a decrease after a peak in the signal intensity due to the removal of the essential oil from the box.

The linalool signal was normalized to the benzene reagent ion, monitored via its isotope (m/Q 79.049873) to avoid saturation of the main reagent peak and scaled by the measured ratio with the benzene (m/Q 78.046410) as is standard for Vocus AIM measurements in benzene mode (Avesani et al., 2026). Inter-box differences were assessed with the paired Friedman test, and the external-contamination excess was computed as the mean of paired differences (external contamination minus baseline) across boxes and runs, with a 95% confidence interval and Wilcoxon signed-rank test.

### 2.3 Validation of kinematic data acquisition pipeline

To validate the kinematic data acquisition pipeline, images were collected continuously for approximately 2 weeks. Stereo image pairs were acquired from all the eight growth boxes at a frame rate of one picture every 3 min (0.00556 Hz).

To validate reliable stereovision between the pair of cameras inside each growth box, we assessed also the time alignment between each stereo pair. Temporal misalignment between stereo cameras introduces apparent disparity errors that are proportional to object motion and focal length. For accurate stereovision, we defined an acceptable maximum apparent displacement of the observed object of Δ *x*_max_ < 0.5 pixels in the acquired images. This threshold ensures that misalignment-induced disparity errors remain below the intrinsic subpixel accuracy of stereo matching algorithms and therefore do not significantly affect depth estimation.

Assuming a binocular geometry with parallel optical axes, the apparent disparity introduced by temporal misalignment can be expressed as follows:

$$\Delta x=\frac{f}{Z}v\text{Δ}t,$$

where *f* is the focal length, *Z* is the depth of the object relative to the camera, *v* is the velocity of the object and Δ *t* is the temporal offset between the two camera acquisitions. From this relationship, the maximum acceptable temporal misalignment Δ *t*_max_ can be derived as:

$$\Delta t_{\text{max}}=\frac{\Delta x_{\text{max}}Z_{\text{min}}}{f_{\max}v_{\text{max}}}.$$

Using conservative parameter values, we set as follows:

- Δ *x*_max_ = 0.5 px;
- *f*_max_ = 2836 px, corresponding to the maximum focal length obtained from camera calibration across all cameras used;
- *Z*_min_ = 500 mm, considering plants positioned in the centre of the growth box and growing to the height where the cameras are located;
- *v*_max_ = 12.13 mm/min, corresponding to the maximum values of velocity reported for *Pisum sativum* in previous studies (Guerra et al., 2019).

Under these assumptions, the resulting maximum acceptable temporal offset is Δ *t*_max_ = 436 ms.

The temporal differences between frames acquired by the left and right cameras of each stereo pair were then computed along the 2 weeks of acquisition.

### 2.4 Validation of the rhizocameras acquisition module

To validate the rhizocameras images acquisition pipeline, data were collected continuously for approximately 2 weeks. Images from rhizo-cameras were acquired from all the eight boxes at a frame rate of one picture every 6 h. Although the system supports higher acquisition frequencies, this rate was chosen to mimic a realistic application that minimizes potential phototropic effects induced by the integrated flashlight, which activates briefly before each image capture. For future applications, the optimal acquisition frequency should be determined based on the specific plant species, experimental objectives and acceptable light exposure levels.

### 2.5 One-day multimodal demo acquisition

To demonstrate the integrated functioning of the Mind(the)Plant facility under fully operational conditions, we report a one-day demonstrative acquisition of a sample box with a single sample plant (*Pisum sativum* L.). The seed was rinsed in tap water for 1 h and germinated in darkness at 24°C (FOC 200IL Connected Cooled Incubator, VELP Scientifica, Usmate, Italy) on filter-paper strips (Whatman 3MM Chr, Cytiva, USA) soaked with demineralized water. After 4 days of germination, the seedling was transplanted into the growth box containing autoclaved silica sand (type 16SS, grain size 0.8–1.2 mm) as substrate. The plant was 11 days old at the start of the demo acquisition. The temperature was set to 22°C with 11 h 15 min photoperiod (5.45 AM to 5 PM). All the modules were run simultaneously, and the acquisition covered a full 24-h period under controlled environmental conditions. For this demo acquisition, we used these sampling frequencies for each module: shoot imaging every 3 min, rhizocamera imaging every 6 h and VOC sampling every ~56 min in multiport mode (7 min for each box), reflecting the different timescales of the processes captured. Raw VOCs data collected with the TOF-MS were processed using an untargeted metabolomics workflow in Tofware software. The workflow comprised the following steps: (i) reference spectrum definition; (ii) peak width and shape refinement; (iii) mass calibration; (iv) peaks identification in a range of m/Q from 30 and 250; and (v) data export. The resulting output consisted of .csv files containing data for each iteration (7 min acquisition with 0.5 Hz sampling rate) in which each row corresponds to a time point and each column represents the intensity of the detected signal. Files obtained were merged using a custom Python module (Python 3.12.3 version) in this way: For each 7-min iteration, the first 2 min and the last 1 min were removed to account for the transition between growth boxes, then the average intensity value was computed. Merging all the iterations, we obtained a time series (one time point every 56 min) for each detected m/Q signal for each growth box. We manually chose the channel associated with monoterpenes (m/Q 136.124741) as representative signal to visualize.

## 3 Results

### 3.1 Validation of VOCs data acquisition pipeline

To assess the uniformity of the eight growth boxes and their isolation from external contamination, we performed five independent alternated acquisition runs in each of two conditions: a baseline condition with no volatile source and an external-contamination condition with a linalool source open inside the phytotron but outside the boxes. Within each run, the multiport valve sequentially sampled all eight boxes, providing five replicate runs per box. For each acquisition, the mean intensity of the linalool ion was computed and normalized to the benzene reagent ion.

The eight boxes did not differ significantly in either condition (Friedman test: baseline *χ*^2^ = 6.47, *p* = 0.49; external contamination *χ*^2^ = 8.20, *p* = 0.32). Inter-box variability was negligible: After normalizing each run to its own mean, the boxes agreed to within a coefficient of variation of ~1%, with a maximum deviation of ~2%. The boxes can therefore be considered equivalent as sampled by the system.

Opening a linalool source outside the boxes raised the in-box signal by 103 normalized ions/s above baseline (paired difference; 95% CI 83–122 normalized ions/s; Wilcoxon signed-rank *p* < 0.001). Although statistically detectable, this excess corresponds to only ~3.9% (95% CI 3.2%–4.6%) of the in-box plateau signal (obtained with the plateau reaching and decay test), defined as the external excess over baseline expressed as a fraction of the mean in-box plateau (measured over the 120 s before source removal). External contamination therefore remains a small fraction of the usable in-box signal (Table 1), confirming that the positive-pressure configuration keeps the boxes effectively isolated under operating conditions. After source removal the plateau reaching and decay test, the in-box signal returned to baseline (within the mean + 3 SD threshold) in 6.6 ± 0.9 min on average (range 4.9–7.6 min across boxes), indicating the time required to clear a box after a saturating exposure.

To account for residual environmental background, each experimental run must include one randomly selected empty box to monitor background levels, which are subsequently subtracted from the signals measured in the remaining boxes.

From the replicated baseline runs, we derived an operational detection threshold for the linalool channel, defined as the mean blank signal plus three standard deviations, equal to ~380 normalized ions/s. Taking the in-box plateau signal (~2620 normalized ions/s) as a reference for the signal at the operating source level, the effective dynamic range between the detection threshold and this reference was ~7×.

**Figure 7:**
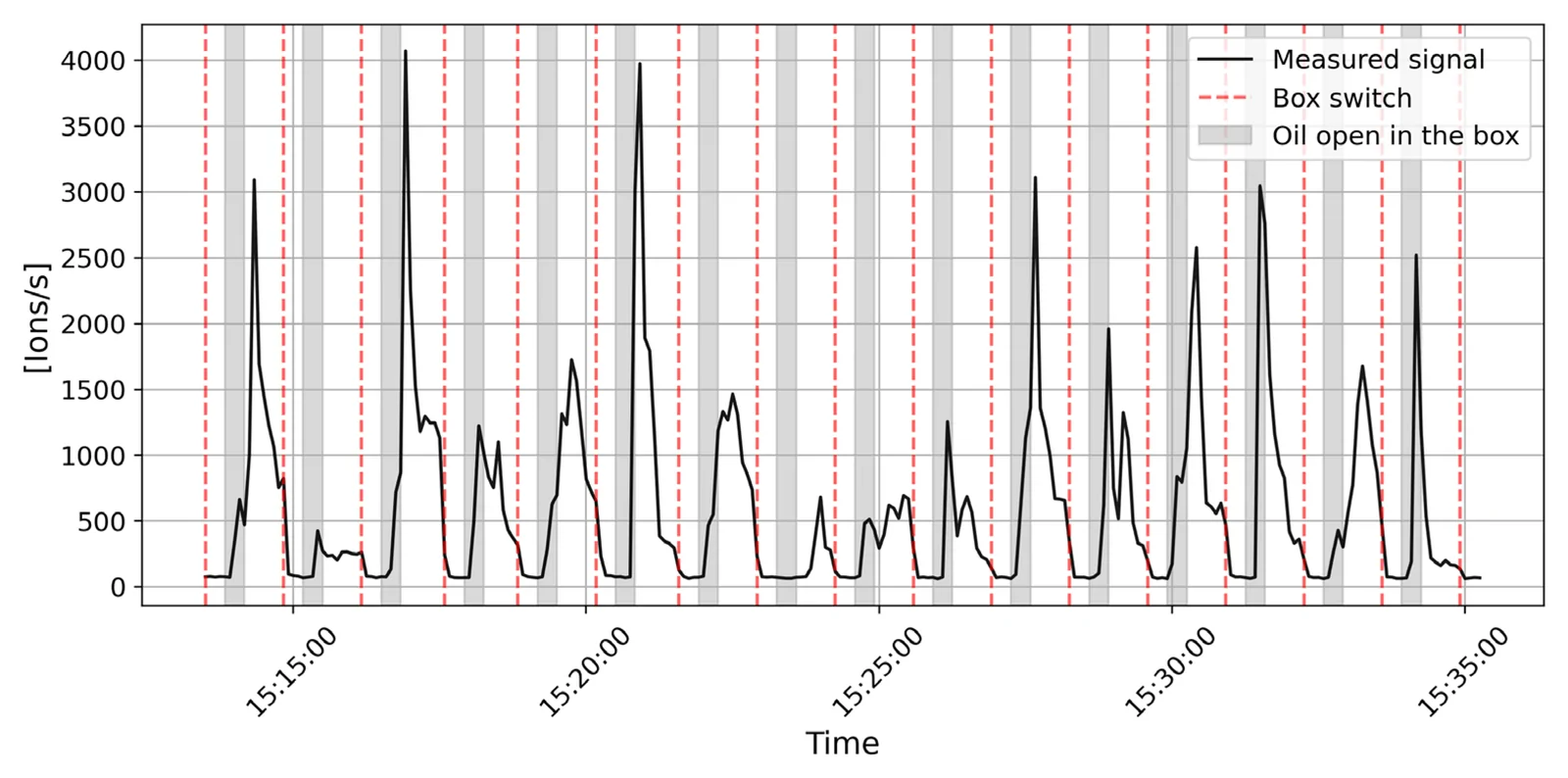
Result of the selective sampling run. Each box has been sampled for a total of 80 s then the multiport switched to the next box. The graph reports two complete runs of acquisitions on all the eight boxes. The black line is the measured signal for linalool. Red dashed lines represent the valve switch to the next box while the grey areas represent the moments when the essential oil containing linalool was open inside the box.

To test that the multiport correctly and selectively samples each box, we analysed the time series collected from the third acquisition run that alternates empty box with linalool signal. Figure 7 shows the trend of the collected signal together with the protocol steps. The expected trend is confirmed so the VOCs acquisition pipeline was considered validated. Figure 7 also shows the absence of significant carry-over effects when the system switches between boxes. Any minimal carry-over dissipates within a few seconds (<10) after valve switching and can be fully mitigated during post-processing by excluding the initial acquisition time window.

### 3.2 Validation of kinematic data acquisition pipeline

No corrupted image files were detected during the entire acquisition period (2 weeks). The expected temporal interval between consecutive frames was 180,000 ms. Across all cameras, the mean observed interval was 180,017, with an average standard deviation (SD) of 1090 ms. As reported in Table 2, the 1st and the 99th percentile differ from the expected value of 180,000 for less than 200 ms, indicating that the vast majority of frame intervals closely matched the nominal acquisition rate while the higher SD is due to a limited number of outliers. In details, the average number of frame intervals differing more than 200 ms from the expected value is 29 which is 0.4% of the total number of frames collected (7043) confirming that deviations from the expected acquisition interval are rare and sporadic.

Analysing the difference between left and right camera in each stereo pair, we obtained a mean time difference absolute value of 4.38 ms (SD 116.31 ms) (Table 3). Values between the 1st and the 99th percentile fell within the acceptable range of ±436 ms, in fact remaining even below ±200 ms.

**Figure 8:**
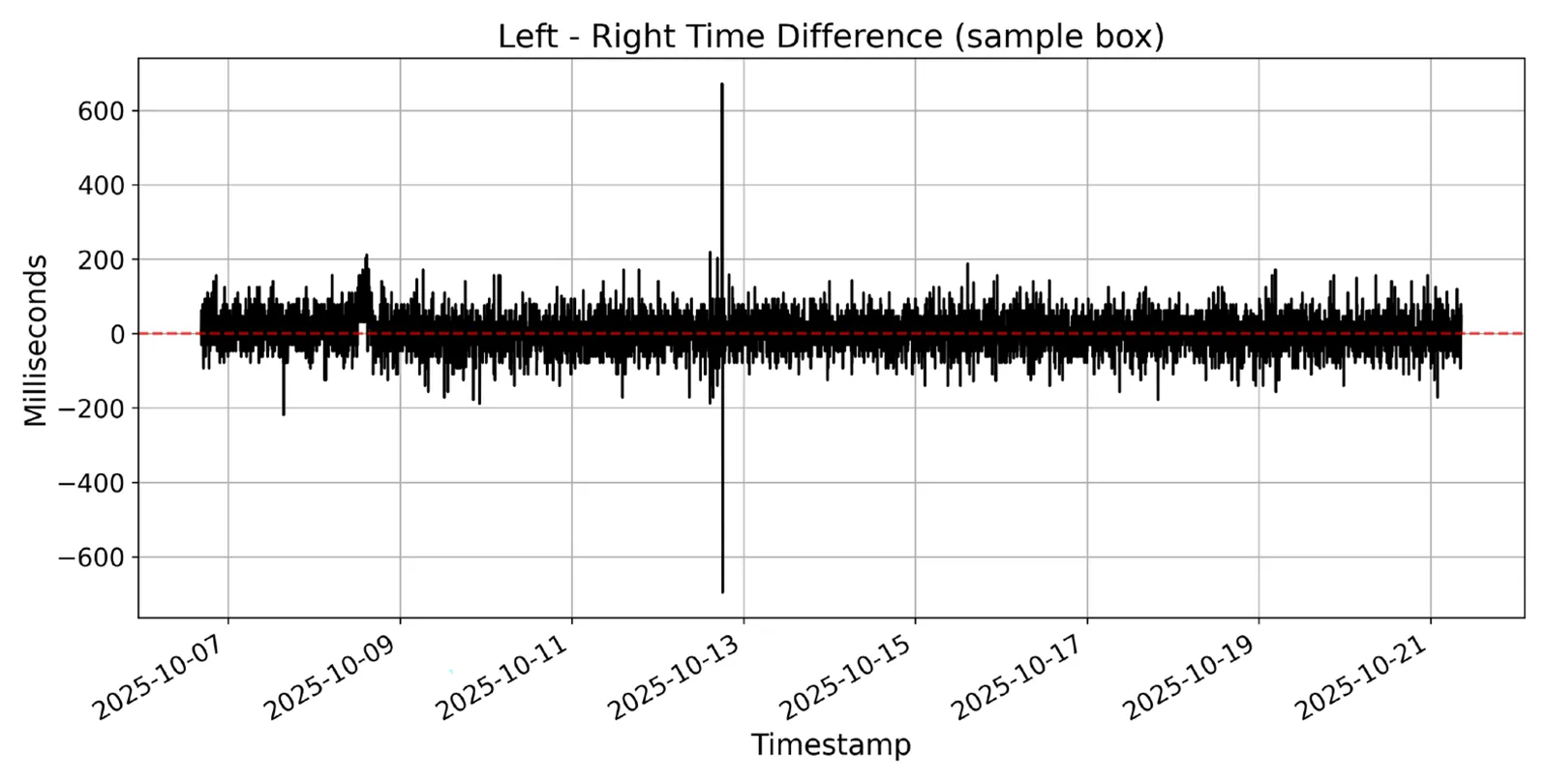
Time series of temporal offset for the stereo pair for a sample box along approximately 2 weeks of acquisition. The isolated spike corresponds to a single momentary acquisition delay of the right camera of the stereo pair, occurring once over the 2-week period and not associated with any recurring system event. It is an example of a sporadic outlier.

On average, only 1.75 samples out of a total of 7044 exhibited an absolute temporal offset exceeding the threshold, corresponding to approximately 0.02% of the total samples and therefore representing rare outlier events probably due to exceptional conditions that can be considered negligible for the intended application, thereby validating the system for reliable stereovision-based kinematic analysis. This is further confirmed by the visual investigation of the trend of temporal offset for a sample stereo pair in one of the boxes (Figure 8).

### 3.3 Validation of the rhizocameras acquisition module

No missing or corrupted images were detected in the collected dataset. Given the nominal inter-frame interval of 6 h, the expected average time difference between consecutive images was 21,600 s. The observed average interval was 21,600.02 s (SD 0.96 s). The minimum and maximum observed intervals were 21,597.46 and 21,602.12, respectively.

These results indicate that the rhizocamera acquisition module operates with high-temporal stability and robustness, validating its suitability for the intended application.

### 3.4 One-day multimodal demo acquisition

To demonstrate the integrated functioning of all acquisition modules—rather than to test a specific biological hypothesis—we report a one-day acquisition of a single sample box where a pea plant was grown. All the modules operated simultaneously, and the acquisition covered a full 24-h period under controlled environmental conditions. Each module sampled at its own frequency (shoot imaging every 3 min, rhizocamera imaging every 6 h and VOC sampling every ~56 min in multiport mode), reflecting the different timescales of the processes captured.

The resulting dataset provides a continuous multimodal representation of plant activity, capturing coordinated fluctuations in movement and VOCs emissions over the diurnal cycle.

**Figure 9:**
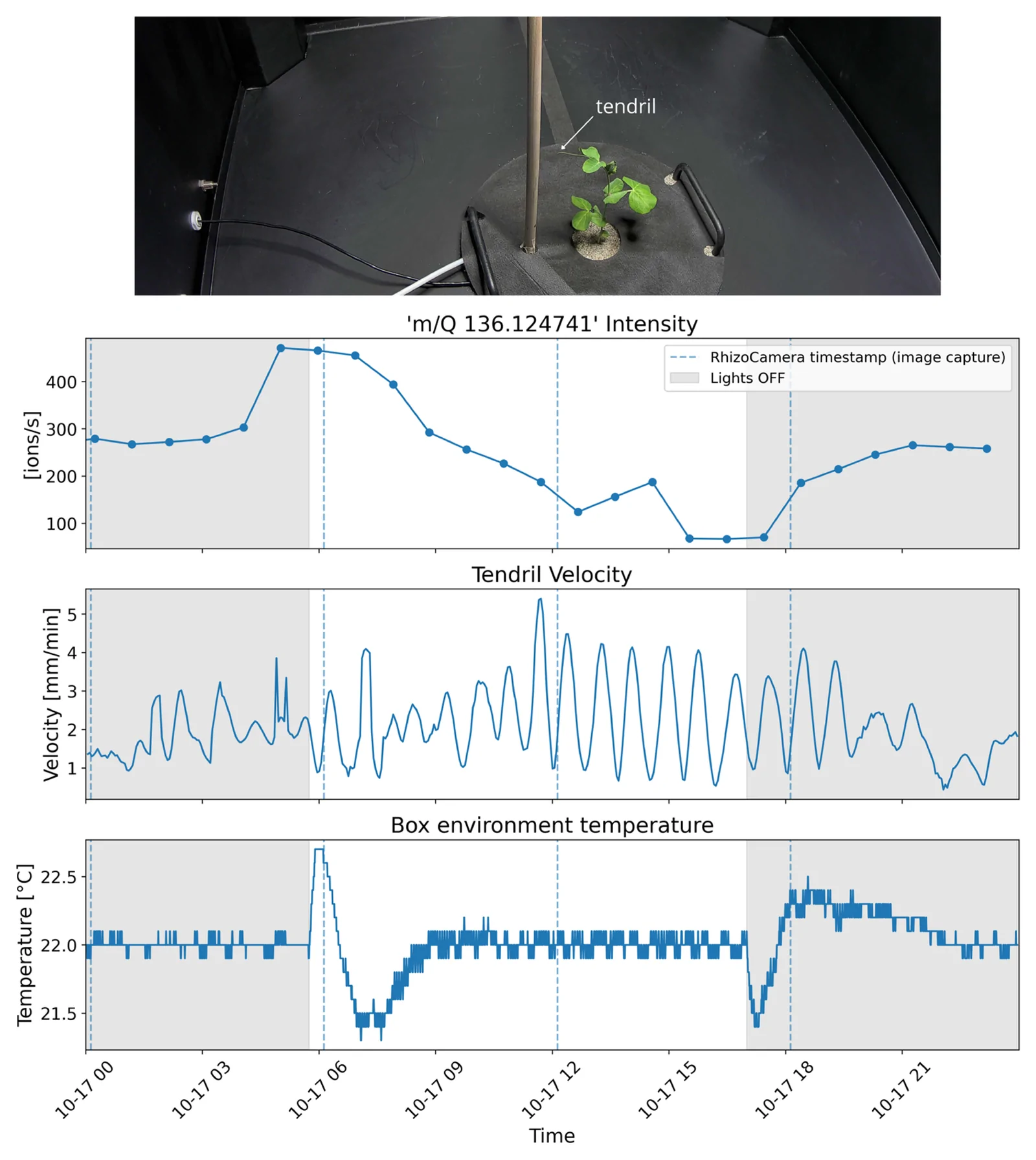
One-day continuous multimodal acquisition. Representative variables showing synchronized multimodal data collection from a single growth box with a pea plant over a 24-h period using the Mind(the)Plant facility. The figure illustrates the temporal alignment of (i) the emission profile of a compound with detected mass associated with monoterpenes (m/Q 136.124741), a group of biogenic VOCs known for their role in plant response to environmental stresses, (ii) shoot (tendril) velocity derived from the three-dimensional trajectory of movement and (iii) box environmental temperature. Below-ground observations obtained from custom rhizocameras at discrete time points are indicated by dashed vertical lines. Shaded areas represent the dark phase of the imposed light–dark cycle.

Visual inspection of the synchronized data (Figure 9) highlights the complementarity of the different modalities: Gradual changes in shoot (tendril) speed can be temporally associated with variations in VOCs emission profiles, while visual inspection of root images provides contextual information on below-ground interactions at key time points (in this specific case no root contact was detected). This integrated acquisition illustrates the capability of the system to capture plant behaviour as a multi-layered process, rather than as a set of isolated measurements.

Overall, the 1-day acquisition further confirms the correct functioning of the infrastructure and demonstrates the feasibility of generating temporally aligned multimodal datasets suitable for integrative analyses of plant behaviour.

## 4 Discussion

The Mind(the)Plant laboratory addresses a key methodological limitation in plant phenomics and behavioural ecology: the lack of experimental platforms enabling synchronized, long-term acquisition of heterogeneous behavioural, morphological and chemical data streams under controlled conditions. While plant movement, VOCs emissions and root growth have been extensively investigated, they are typically measured using independent experimental setups, limiting temporal alignment and integrative analyses. By integrating these modalities within a single infrastructure, the system enables temporally aligned multimodal analyses that are not achievable with isolated measurement approaches, providing a robust methodological foundation for the study of plant behaviour as an integrated process.

The validation tests demonstrate that all acquisition modules operate reliably over multi-week recordings, with stable performance, low failure rates and minimal data loss. Importantly, the system ensures precise temporal synchronization across modalities, enabling analyses that move beyond single-trait approaches toward integrated investigation of plant behaviour. From an ecological perspective, this capability supports more comprehensive controlled and reproducible studies of dynamic plant–plant and plant–environment interactions, including neighbour detection, resource competition and stress-related signalling processes. Because the volatile headspace is shared within a box, studies on social dynamics must be designed at the level of the group of plants, comparing experimental conditions with matched plant numbers under different manipulations.

Beyond hardware integration, another contribution of the Mind(the) Plant facility lies in its data architecture and processing pipelines. In line with best practices in digital and computational phenomics (Harfouche et al., 2023), the system ensures traceability from raw data to processed outputs and facilitates reproducible multimodal analyses. By integrating imaging, chemical and environmental data within a unified framework, the facility reduces technical variability and enhances comparability across experiments, supporting scalable data reuse and cross-study analyses. A specific analytical challenge in exploiting these datasets arises from the different temporal scales of the modalities. The temporal synchronization ensured by the facility provides the common time base on which their integration relies: Data streams can be aggregated or resampled onto shared temporal grids, with features extracted on common temporal windows. Cross-scale relationships can then be examined using multi-scale time-series methods (e.g. wavelet-based analysis, lagged cross-correlation), while multivariate techniques such as machine-learning based approaches can help identify emergent, whole-plant patterns across modalities. These data-driven analyses are enabled by, but distinct from, the acquisition facility itself: Their capacity to reveal robust or causal structure ultimately depends on the experimental design.

While the current configuration focuses on kinematics, VOCs and root imaging, the system has been designed to incorporate electrophysiological recordings to further expand the range of measurable plant responses. Future implementations will integrate electrode-based acquisition systems within each growth box to collect electrophysiological signals from the aerial part of the plants, following approaches used in plant electrome studies (de Carvalho Oliveira et al., 2025; Parise et al., 2021), enabling synchronized recording of electrical signals alongside other modalities. Each growth box is already equipped with a dedicated power supply inlet, ensuring stable in situ operation. We plan to explore smart acquisition systems (e.g. Raspberry Pi-based solutions) connected via Ethernet, enabling live data streaming and direct storage of raw signals within the laboratory data lake. The integration of these signals will further strengthen the capacity of the platform to investigate plant behaviour as a distributed and dynamic process.

The modular design of the system allows flexible extension of its components. A key limitation of the current configuration concerns the characterization of root dynamics, which is presently restricted to localized observations obtained through custom rhizocameras. Future developments could incorporate transparent growth media, such as water or agar, combined with dedicated stereovision systems, enabling full three-dimensional reconstruction of root architecture and movement, as proposed in previous work (Simonetti et al., 2024). Such developments would enhance the ability to study coordinated above- and below-ground processes in terms of root architecture and motion. However, such media are envisaged specifically for root imaging because below-ground VOCs sampling in the current system draws from the gaseous headspace of the pot compartment, which is absent when roots are embedded in a hydrogel; this configuration would provide full 3D characterization of root motion while restricting VOCs sampling to the above-ground compartment.

It is also important to acknowledge the boundaries of the signalling channels that the current configuration can capture. Recent studies report that plant behaviour and communication rely on a broad repertoire of chemical and physical signals including, beyond VOCs, small RNAs, plant-emitted ultrasound, and hydraulic, electrical and contact-mediated cues (Dobránszki et al., 2025), many of which require dedicated sensing principles. The present implementation therefore captures only a subset of the signals through which plants interact with their environment and with one another. The current platform should therefore be understood as a foundational framework rather than an exhaustive solution. Its modular architecture is designed to accommodate future expansions, and the integration of additional sensor types or acquisition modules will be necessary as the field advances toward a more complete characterization of plant behaviour. In addition, further studies across different taxa and environmental conditions will be necessary to assess the generality and ecological relevance of the approach.

A limitation of the current setup is that the VOCs monitoring module relies on direct-injection high-resolution TOF-MS without chromatographic separation. This entails a specific trade-off. On the one hand, accurate-mass measurements allow molecular formulae to be assigned, and the absence of a chromatographic step enables monitoring of the volatile bouquet at high-temporal resolution, which is essential for relating emission dynamics to the other modalities. On the other hand, isomeric and isobaric compounds cannot be resolved by mass alone. The system is therefore suited to tracking the temporal dynamics of volatile emission at the level of accurate-mass channels, rather than to the exhaustive identification of the VOCs bouquet. For compound identification, targeted offline sampling (e.g. sorbent tubes or SPME) followed by GC–MS provides a complementary annotation step, after which identified compounds can be monitored continuously by the online system.

In terms of adoption, a facility such as Mind(the)Plant entails non-negligible financial and operational costs. The implementation of high-resolution imaging systems, mass spectrometry instrumentation and dedicated IT infrastructure requires substantial initial investment, as well as ongoing maintenance to ensure data quality and system stability. In addition, the operation of the platform requires multidisciplinary expertise spanning plant biology, analytical chemistry, bioinformatics and software development. However, the modular architecture of the system partially mitigates these limitations by enabling scalable adoption. Cost-effective configurations can be achieved by reducing the number of growth boxes or by implementing selected modules. Furthermore, individual components can be replaced with lower-cost alternatives suitable for proof-of-concept studies or applications with less demanding performance requirements. The mass spectrometry unit represents the main cost driver of the system and, in some contexts, could be substituted with more affordable technologies such as electronic noses (Laothawornkitkul et al., 2008). Although these alternatives provide lower sensitivity and reduced capabilities for untargeted analysis, they may still be appropriate for specific experimental objectives. Overall, this flexibility promotes broader accessibility and facilitates adoption across laboratories with different levels of resources.

Taken together, the Mind(the)Plant facility enables the study of plant behaviour as an integrated dynamical system, moving beyond isolated measurements toward coordinated, multi-scale analyses of plant responses. By providing a unified framework for the simultaneous monitoring of multiple signalling modalities, the platform opens new possibilities for investigating plant behaviour, signalling and adaptation at the interface of plant ecology, behaviour and evolution.

## 5 Scientific Questions and Investigation Possibilities

The Mind(the)Plant facility provides a unique experimental framework for the systematic investigation of plant behaviour by integrating multimodal monitoring of shoots, roots and VOCs emissions. The facility enables controlled, reproducible studies of plant responses to internal and external stimuli, allowing for a broad range of scientific questions to be addressed. We emphasize that the facility provides the means to deliver controlled, temporally structured stimuli and to record plant responses in a synchronized manner; addressing questions such as those below depends on the experimental design implemented with the platform rather than on the platform alone.

### 5.1 Behavioural adaptation and response

The facility allows precise quantification of plant growth and movement under controlled environmental conditions. Key questions that can be addressed include:

- How are shoot kinematics and root growth dynamics temporally coordinated under controlled environmental perturbations (e.g. drought, nutrient stress, temperature or light manipulation) or in response to other experimental manipulations such as controlled VOCs exposure or different climbing supports (when investigating climbing plants)?
- Using appropriate experimental designs (e.g. combining programmed environmental sequences using the PLC, presenting supports with different mechanical properties) and control conditions, can we reveal anticipatory mechanisms and decision-making processes in kinematic modulation and VOCs emission?

### 5.2 Plant signalling and communication

The VOCs monitoring capabilities facilitate the study of plant chemical signalling and communication. Potential investigations include:

- How do VOCs emission patterns vary temporally and between compartments (above- and below-ground) in response to environmental cues or stressors?
- Can changes in VOCs profiles predict subsequent morphological or physiological responses in the same plant or in neighbouring individuals?

### 5.3 Integrated system-level behaviour

The combined measurement of kinematics, VOCs and root growth enables exploration of whole-plant behaviour at the system level. Examples of research questions can include:

- How are above-ground (shoot) and below-ground (root) growth coordinated under varying environmental or chemical conditions?
- Can correlations among motion, VOC emissions and root dynamics reveal emergent patterns of plant behaviour or adaptation strategies?

Its combined features position Mind(the)Plant as a versatile system for advancing our understanding of plant behaviour, signalling and adaptation, enabling studies that link morphological, chemical and potentially electrophysiological responses in an integrated framework, in this way capturing traits that emerge from coordinated whole-plant responses rather than single measurements.

## Figures and Tables

**Table 1:** Inter-box VOC validation summary.

Box	Baseline mean (norm ions/s)	Contamination mean (norm ions/s)	Delta (contam-baseline)	Plateau (norm ions/s)	Contamination (% of plateau)	Recovery to baseline (min)
1	287.3 (SD 34.1)	378.9 (SD 89.2)	91.6	3106.1	2.9	7.2
2	288.3 (SD 35.8)	387 (SD 90.6)	98.8	2413.2	4.1	7.6
3	284.7 (SD 34.1)	386.9 (SD 89)	102.2	3201.8	3.2	6.2
4	281.2 (SD 36.5)	386.7 (SD 84.1)	105.5	2622.8	4	7.1
5	283.7 (SD 35.8)	384.7 (SD 83.3)	101	2637.5	3.8	6.7
6	282.9 (SD 36.3)	383.1 (SD 81.8)	100.2	2412.7	4.2	6
7	280.1 (SD 33.9)	387.4 (SD 84.1)	107.3	2696.7	4	7
8	277.6 (SD 37.1)	392.1 (SD 85)	114.5	1868.6	6.1	4.9

*Note*: For each of the eight growth boxes, mean linalool signal under baseline (no volatile source) and external-contamination conditions (linalool source open inside the phytotron but outside the boxes), each averaged over five independent acquisition runs (mean and SD). Delta is the external-contamination excess over baseline. Plateau is the in-box reference signal measured during the plateau reaching and decay test (mean over the 120 s before source removal); Contamination % of plateau expresses the external excess as a fraction of this reference. Recovery to baseline is the time for the signal to return within the detection threshold (mean + 3 SD of baseline) after source removal.

**Table 2:** Descriptive statistics of the time gaps in milliseconds (ms) between collected images from each camera.

Camera	Total samples	Number of gaps >180,200 or <179,800	Mean (ms)	SD (ms)	P01 (ms)	Median (ms)	P99 (ms)
box1_left	7043	23	180,017	780	179,902	180,018	180,132
box1_right	7043	26	180,017	1079	179,905	180,017	180,134
box2_left	7043	26	180,017	1081	179,901	180,018	180,137
box2_right	7043	27	180,017	1080	179,899	180,017	180,131
box3_left	7043	29	180,017	1221	179,899	180,017	180,130
box3_right	7043	30	180,017	1212	179,898	180,017	180,140
box4_left	7043	31	180,017	1172	179,904	180,017	180,133
box4_right	7043	35	180,017	1171	179,901	180,017	180,135
box5_left	7043	28	180,017	1120	179,903	180,017	180,129
box5_right	7043	28	180,017	1119	179,903	180,016	180,139
box6_left	7043	30	180,017	1115	179,905	180,016	180,127
box6_right	7043	32	180,017	1114	179,905	180,017	180,134
box7_left	7043	33	180,017	1067	179,897	180,018	180,129
box7_right	7043	34	180,017	1067	179,897	180,016	180,139
box8_left	7043	28	180,017	1018	179,903	180,018	180,130
box8_right	7043	30	180,017	1018	179,905	180,016	180,126
Mean	7043	29	180,017	1090	179,902	180,017	180,133

*Note*: Columns reported are (from left to right): Camera identifier, total images collected, number of time gaps differing more than 200 ms from the expected value, mean, standard deviation, minimum value, 1st percentile, median, 99th percentile, maximum value.

**Table 3:** Descriptive statistics of the temporal offsets in milliseconds (ms) between left and right camera of each stereo pair.

Box	Total samples	Number of samples above threshold	Mean (ms)	SD (ms)	P01 (ms)	Median (ms)	P99 (ms)
boxl	7044	4	−25.72	529.92	−140.62	−15.63	109.37
box2	7044	3	3.82	46.88	−109.38	0.00	125.00
box3	7044	3	5.72	115.99	−109.38	0.00	125.01
box4	7044	0	4.09	49.55	−124.99	0.00	124.99
box5	7044	l	−18.40	51.24	−156.26	−15.62	93.76
box6	7044	l	−11.61	44.10	−125.00	−15.62	93.77
box7	7044	0	0.10	45.45	−124.98	0.00	109.38
box8	7044	2	6.97	47.34	−109.38	0.00	124.99
Mean	7044	1.75	−4.38	116.31	−125.00	−5.86	113.28

*Note*: Columns reported are (from left to right): Box identifier, total samples per camera, number of samples above the threshold of ±436 ms for good stereovision, mean, standard deviation, minimum value, 1st percentile, median, 99th percentile, maximum value.

## Data Availability

Data, code and processing pipelines supporting this study are available at https://doi.org/10.5281/zenodo.22095454 (Simonetti & Castiello, 2026).
